# Efficacy of the CDK4/6 Dual Inhibitor Abemaciclib in EGFR-Mutated NSCLC Cell Lines with Different Resistance Mechanisms to Osimertinib

**DOI:** 10.3390/cancers13010006

**Published:** 2020-12-22

**Authors:** Silvia La Monica, Claudia Fumarola, Daniele Cretella, Mara Bonelli, Roberta Minari, Andrea Cavazzoni, Graziana Digiacomo, Maricla Galetti, Francesco Volta, Maicol Mancini, Pier Giorgio Petronini, Marcello Tiseo, Roberta Alfieri

**Affiliations:** 1 Department of Medicine and Surgery, University of Parma, 43126 Parma, Italy; silvia.lamonica@unipr.it (S.L.M.); claudia.fumarola@unipr.it (C.F.); daniele.cretella@unipr.it (D.C.); mara.bonelli@unipr.it (M.B.); andrea.cavazzoni@unipr.it (A.C.); graziana.digiacomo@unipr.it (G.D.); francesco.volta@unipr.it (F.V.); piergiorgio.petronini@unipr.it (P.G.P.); 2 Medical Oncology Unit, University Hospital of Parma, 43126 Parma, Italy; rominari@ao.pr.it; 3 Italian Workers’ Compensation Authority (INAIL) Research Center, 43126 Parma, Italy; m.galetti@inail.it; 4 Cancer Research Institute of Montpellier (IRCM), CEDEX 5, 34298 Montpellier, France; maicol.mancini@inserm.fr

**Keywords:** non-small cell lung cancer, osimertinib, resistance, epidermal growth factor receptor, abemaciclib, CDK4/6 inhibitors

## Abstract

**Simple Summary:**

Osimertinib, a third-generation irreversible epidermal growth factor receptor (EGFR) tyrosine kinase inhibitor (TKI), has shown marked clinical benefit for non-small cell lung cancer (NSCLC) patients with EGFR activating mutations. However, resistance to osimertinib inevitably develops and heterogeneous mechanisms of acquired resistance have been documented. Therefore, new strategies to bypass resistance are urgently needed. In this study, we investigated the potential activity of abemaciclib as second-line therapeutic approach after osimertinib progression and the effect of combining abemaciclib with osimertinib on the appearance of resistance in osimertinib-sensitive models.

**Abstract:**

Abemaciclib is an inhibitor of cyclin-dependent kinases (CDK) 4 and 6 that inhibits the transition from the G1 to the S phase of the cell cycle by blocking downstream CDK4/6-mediated phosphorylation of Rb. The effects of abemaciclib alone or combined with the third-generation epidermal growth factor receptor (EGFR) tyrosine kinase inhibitor (TKI) osimertinib were examined in a panel of PC9 and HCC827 osimertinib-resistant non-small cell lung cancer (NSCLC) cell lines carrying EGFR-dependent or -independent mechanisms of intrinsic or acquired resistance. Differently from sensitive cells, all the resistant cell lines analyzed maintained p-Rb, which may be considered as a biomarker of osimertinib resistance and a potential target for therapeutic intervention. In these models, abemaciclib inhibited cell growth, spheroid formation, colony formation, and induced senescence, and its efficacy was not enhanced in the presence of osimertinib. Interestingly, in osimertinib sensitive PC9, PC9T790M, and H1975 cells the combination of abemaciclib with osimertinib significantly inhibited the onset of resistance in long-term experiments. Our findings provide a preclinical support for using abemaciclib to treat resistance in EGFR mutated NSCLC patients progressed to osimertinib either as single treatment or combined with osimertinib, and suggest the combination of osimertinib with abemaciclib as a potential approach to prevent or delay osimertinib resistance in first-line treatment.

## 1. Introduction

Osimertinib is the first mutant-selective third-generation epidermal growth factor receptor (EGFR) tyrosine kinase inhibitor (TKI) that has been approved for patients with EGFR oncogene-addicted advanced non-small cell lung cancer (NSCLC) [1]. Osimertinib is the only approved third-generation EGFR-TKI for T790M-positive patients progressed on first- or second generation EGFR-TKIs [2,3]; moreover, it has recently been approved as first-line therapy for advanced EGFR-mutated NSCLC [4], considering the benefit in progression-free survival (PFS) and overall survival (OS) as compared with gefitinib or erlotinib evidenced in the FLAURA trial [5,6].

Despite the efficacy of osimertinib in first- and second-line EGFR mutated NSCLC settings, patients inevitably develop acquired resistance and chemotherapy remains the only therapeutic option for these patients [4]. Differently from resistance mechanisms developed after gefitinib/erlotinib treatment, which are mainly associated with the acquisition of T790M mutation in EGFR exon 20, the acquired osimertinib resistance is highly heterogeneous, including EGFR-dependent and -independent mechanisms [7]. EGFR C797, G796, L792, L718/G719 and G724 mutations, EGFR gene amplification, MET and HER2 amplification, BRAF, RAS-MAPK and PI3K pathway activation, oncogenic fusion mutations and phenotypic transformation are the emerging resistance mechanisms to osimertinib in EGFR-mutated NSCLC patients. Some of these mechanisms have been found to confer resistance to osimertinib after either first- or second-line therapy, while others seem exclusive to one type of setting [7]. The high heterogeneity and the coexistence of multiple resistance mechanisms in the same patient represent a major challenge in the treatment of osimertinib-resistant patients.

Abemaciclib (LY835219) is an orally available, small molecule inhibitor of cyclin-dependent kinases (CDK) 4 and 6 that blocks the transition from the G1 to the S phase of the cell cycle with consequent growth arrest. Based on the data from MONARCH-1–2–3 trials, abemaciclib received FDA approval as monotherapy or in combination with fulvestrant or with an aromatase inhibitor for hormone receptor (HR)-positive, human epidermal growth factor receptor 2 (HER2)-negative metastatic breast cancer patients [8,9,10]. Beyond the inhibition of cell proliferation, all the FDA approved CDK4/6 inhibitors exert other effects on cancer cells and tumor microenvironment, as recently reviewed by our group [11]. The combination of CDK4/6 inhibitors with EGFR inhibitors has been tested in few preclinical studies so far and deserves further investigation. One study evaluated the effects of palbociclib combined with osimertinib only in one osimertinib-resistant cell model (H1975) with an undefined mechanism of resistance [12]. In another paper, palbociclib was used in combination with afatinib in two afatinib-resistant cell models that were not characterized for the underlying mechanisms of resistance [13].

Here we investigated the potential efficacy of abemaciclib in a panel of PC9, PC9T790M, and HCC827 resistant cells with EGFR-dependent or –independent, intrinsic or acquired mechanisms of resistance. We explored the status of the CDK4/6-cyclin D-Rb pathway, and analyzed the effects of the CDK4/6 inhibitor either alone or combined with osimertinib on cell proliferation, colony formation, growth of spheroids, cell death, and cell senescence. Moreover, we tested the efficacy of combining abemaciclib and osimertinib in preventing the acquisition of resistance in osimertinib sensitive cell models.

## 2. Results

### 2.1. Osimertinib-Resistant Cell Models Up-Regulate the Phosphorylation of Rb Protein and Display Sensitivity to CDK4/6 Inhibition

Firstly, we tested the phosphorylation and expression of the cell cycle related proteins Rb, Cyclin D1, and p16^INK4a^ in a panel of osimertinib-resistant NSCLC cells originated from EGFR-mutated PC9, PC9T790M, and HCC827 cell lines.

**Figure 1:**
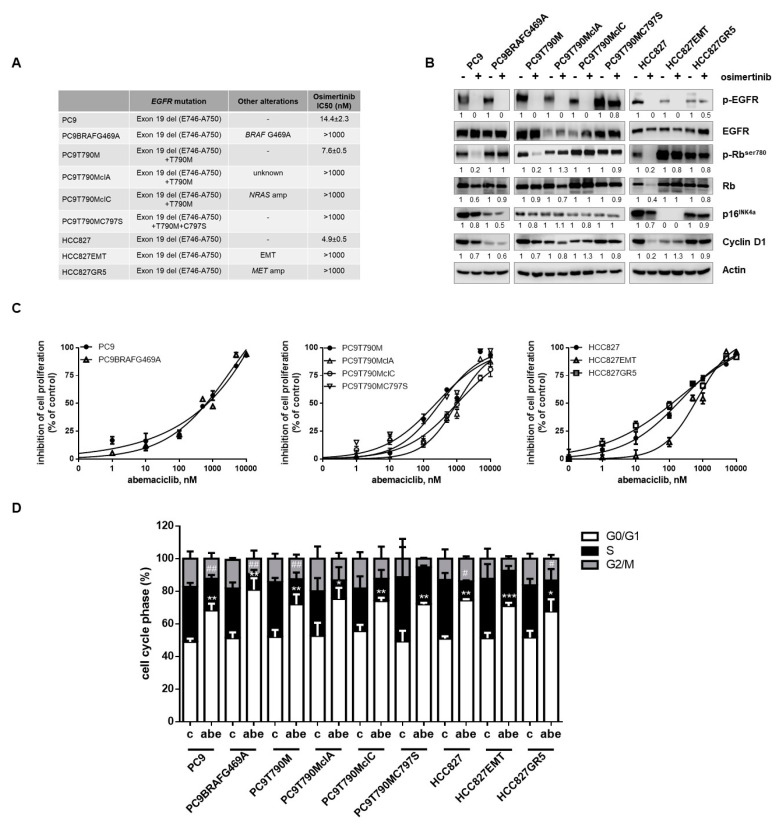
Abemaciclib sensitivity of NSCLC cells resistant to osimertinib. (**A**) Characteristics of NSCLC osimertinib-resistant cell lines used in this study. (**B**) Osimertinib-sensitive and resistant cells were treated with osimertinib (50 nM and 500 nM, respectively) and after 24 h the expression level of p-Rb^ser780^, Rb, p16^INK4A^ and Cyclin D1 were evaluated by Western blotting. Results are representative of at least two independent experiments. (**C**) The indicated cell lines were treated with increasing concentrations of abemaciclib for 6 days. Cell proliferation was assessed by MTT assay; for each cell model, the data are expressed as a percentage of inhibition vs. the corresponding untreated control cells and are means ± standard deviation (SD) of three independent experiments. (**D**) The indicated cell lines were treated with 500 nM abemaciclib for 24 h. Then, the cells were stained with propidium iodide and their distribution in cell cycle phases was determined by flow cytometry. Results are means ± SD of three independent experiments. * *p* < 0.05, ** *p* < 0.01, *** *p* < 0.001 vs. G0/G1 phase of corresponding control cells; # *p* < 0.05, ## *p* < 0.01, vs. S phase of corresponding control cells).

The resistant clones tested showed different mechanisms of intrinsic or acquired resistance to osimertinib, as described in the Materials and Methods Section (Section 4) and reported in Figure 1A.

Independently of the resistance mechanisms, all osimertinib-resistant NSCLC cell clones maintained a higher phosphorylation of Rb in the presence of osimertinib when compared with the parental sensitive cell lines (Figure 1B); p-EGFR was inhibited in all cells with an EGFR-independent mechanism of resistance; only in PC9T790MC797S cells, carrying the triple mutated EGFR, osimertinib failed to inhibit phosphorylation. Cyclin D1 expression was downregulated by osimertinib in the parental cells, whereas was only slightly affected in the cell clones. Interestingly, only HCC827EMT osimertinib-resistant cells were negative for the expression of the cell cycle inhibitor p16^INK4a^, while all the other cells, either sensitive or resistant, expressed this protein. We then tested the responsiveness to abemaciclib in terms of cell proliferation inhibition and cell cycle arrest. All the resistant cell lines showed sensitivity to abemaciclib, with IC_50_ values ranging from 200 nM to 1 µM, comparable to those of the respective parental cells (Figure 1C). As expected, the treatment with abemaciclib increased the fraction of cells in the G0/G1 phase and decreased the fraction in the S-G2/M phase in both sensitive and resistant cells (Figure 1D).

### 2.2. Effects of Abemaciclib Alone or Combined with Osimertinib on 2-D and 3-D Cell Growth and on the Expression of Cell Cycle-Related Proteins

**Figure 2:**
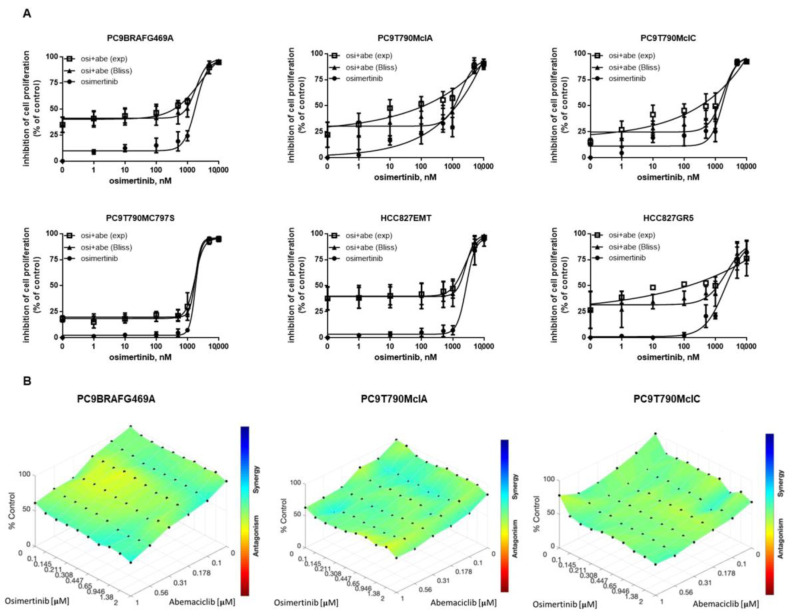
Effects of the combined treatment of abemaciclib with osimertinib on cell proliferation. (**A**) The indicated cells were treated with different concentrations of osimertinib in absence or in presence of 500 nM abemaciclib. After 72 h cell proliferation was assessed by MTT assay and the effect of the drug combination was evaluated using the Bliss interaction model. Data are expressed as percent inhibition vs. control cells and are means ± SD of at least three separate experiments. (**B**) Drug interaction heatmaps for osimertinib and abemaciclib combination treatment in PC9BRAFG469A, PC9T790MclA, and PC9T790MclC cells.

We then evaluated whether treatment with abemaciclib could restore sensitivity to osimertinib in the resistant clones. The concomitant treatment of osimertinib with a fixed concentration of abemaciclib did not induce an enhanced inhibition of cell viability as evaluated by the Bliss analysis (Figure 2A), indicating that the reduced viability was mainly ascribed only to abemaciclib treatment, at least up to 1 µM osimertinib, a concentration higher than plasma level in patients treated with 80 mg/day dosage [14]. To better investigate the nature of the interaction between abemaciclib and osimertinib, we tested multiple drug concentrations, and the data, represented as surface matrix plot (Figure 2B), confirmed the absence of synergy between the two drugs. We performed additional experiments with palbociclib and despite this CDK4/6 inhibitor was less effective than abemaciclib, the obtained results were comparable to those with abemaciclib (Figure S1).

**Figure 3:**
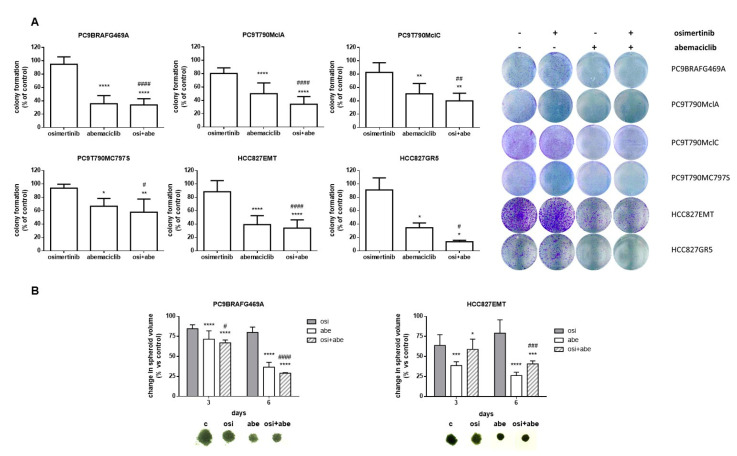
Effects of abemaciclib alone or combined with osimertinib on colony formation and 3-D cell growth. (**A**) The indicated osimertinib resistant cells were treated with 500 nM osimertinib, 500 nM abemaciclib or their combination and after 6 days colony formation was assessed as described in the Materials and Methods section. Representative images of crystal violet staining of colonies are shown. Data are the means ± SD of at least three independent experiments. (**B**) The growth of spheroids from PC9BRAFG469A and HCC827EMT cells was analyzed after 3 and 6 days of treatment with abemaciclib, osimertinib or both drugs. The data are expressed as percent of spheroid growth versus control. Representative images of spheroids after 6 days of culture are shown. Data are representative of two independent experiments (* *p* < 0.05, ** *p* < 0.01, *** *p* < 0.001, **** *p* < 0.0001 vs. control; # *p* < 0.05, ## *p* < 0.01, ### *p* < 0.001, #### *p* < 0.0001 vs. osimertinib).

By a cell colony formation assay, we confirmed the efficacy of either abemaciclib alone or abemaciclib combined with osimertinib in reducing the number of colonies after 6 days of exposure to the drugs in all resistant cell models (Figure 3A) without statistical difference between the two conditions.

We then generated tumor spheroids from PC9BRAFG469A and HCC827EMT cells and demonstrated that abemaciclib treatment significantly reduced the volume of tumor spheroids (Figure 3B). We did not observe any difference between abemaciclib and the combined treatment, again indicating the effectiveness of abemaciclib alone. Our results indicate that treatment with abemaciclib did not restore sensitivity to osimertinib in the resistant clones but exerted a strong activity in reducing their proliferation as single drug.

We then evaluated the effect of osimertinib, abemaciclib or their combination on the expression and activation of cell cycle-related proteins in both osimertinib-resistant and sensitive parental cells. Rb and CDK6 phosphorylation were strongly inhibited by osimertinib alone in PC9, PC9T790M, and in HCC827 cells as expected, being these cell lines highly responsive to osimertinib. 

**Figure 4:**
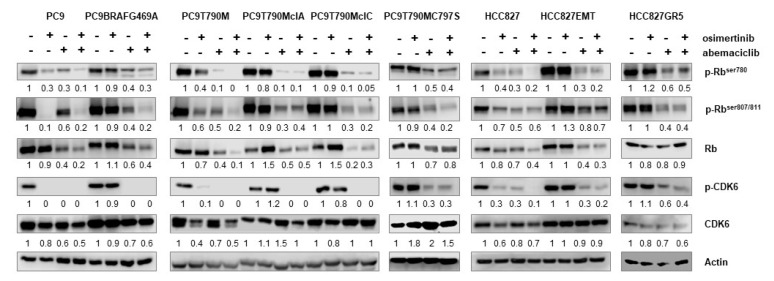
Effect of abemaciclib alone or combined with osimertinib on cell cycle proteins in NSCLC osimertinib resistant cell lines. Cells were treated with osimertinib (50 nM for sensitive and 500 nM for resistant cells), abemaciclib (500 nM) or their combination and after 24h were lysed and western blot analysis was performed to detect the indicated proteins. Results are representative of at least two independent experiments.

In all the resistant models, osimertinib failed to downregulate the phosphorylation of these proteins, but abemaciclib alone or combined with osimertinib efficaciously decreased p-Rb and p-CDK6 levels, mimicking the effect of osimertinib alone in parental sensitive cells (Figure 4).

### 2.3. Effects of Abemaciclib Alone or Combined with Osimertinib on Cell Death and Senescence

**Figure 5:**
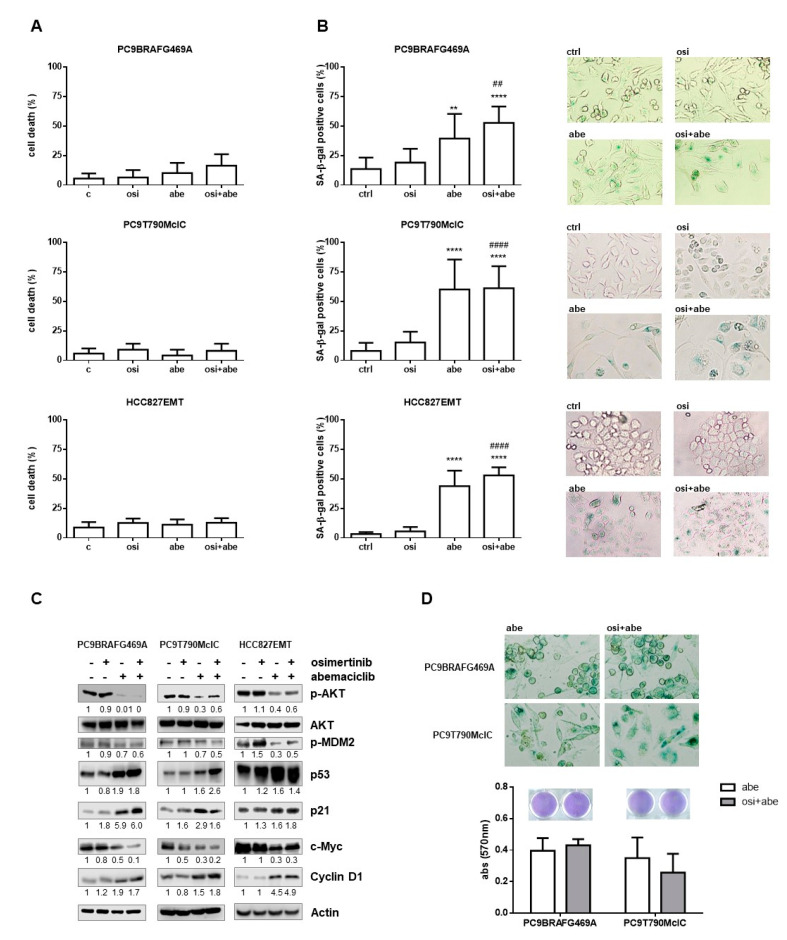
Effect of abemaciclib alone or combined with osimertinib on cell death and senescence in NSCLC osimertinib resistant cell lines. Cells were treated with 500 nM osimertinib, 500 nM abemaciclib or their combination for 72 h. (**A**) Cell death was quantified by fluorescence microscopy analysis on Hoechst 33342 and propidium iodide-stained cells. Data are representative of two independent experiments. (**B**) Senescent cells were quantified by SA-β-Gal staining. Histograms represent the percentage of senescent cells positive for SA-β-Gal expression and are means ± SD of data from three independent experiments. (** *p* < 0.01, **** *p* < 0.0001 vs. control; ## *p* < 0.01, #### *p* < 0.0001 vs. osimertinib). (**C**) The cells were lysed and western blot analysis was performed to detect the indicated proteins. Results are representative of two independent experiments. (**D**) PC9BRAFG469A and PC9T790MclC cells were treated with 500 nM abemaciclib in absence or presence of 500 nM osimertinib. After 4 weeks senescence was assessed by β-galactosidase staining and cell proliferation was evaluated by crystal violet staining. Columns are the means ± SD of 12 replicates. In (**B**) and (**D**), representative images of SA-β-Gal-stained cells are shown (magnification 100×).

We evaluated the effect of drug treatments on cell death induction in PC9BRAFG469A, PC9T790MclC, and HCC827EMT cells. Abemaciclib as well as palbociclib elicit cytostatic more than cytotoxic responses, even if abemaciclib was reported to be substantially more efficacious than palbociclib in killing p-Rb-proficient breast cancer cells [15]. Differently, in PC9BRAFG469A, PC9T790MclC, and HCC827EMT cell resistant clones, either abemaciclib or the combined treatment failed to induce significant cell death (Figure 5A). These results indicate that induction of cell death is not relevant for the effectiveness of abemaciclib.

Considering that beyond the inhibition of cell proliferation, the CDK4/6 inhibitors may exert other effects on cancer cells such as the induction of a senescent-like phenotype [11], we tested the production of the Senescent-Associated β-galactosidase enzyme (SA-β-Gal) in these resistant cell models. As shown in Figure 5B, abemaciclib induced senescence in PC9BRAFG469A, PC9T790MclC, and HCC827EMT cells with a percentage of senescent cells, characterized by enlarged morphology, around 40–50% after 3 days of treatment. This percentage was not further increased by the combined treatment, confirming the strong efficacy of abemaciclib alone. To gain insights into the molecular mechanism by which abemaciclib induced senescence in the resistant clones, we analyzed the level of proteins critical for senescence regulation in PC9BRAFG469A, PC9T790MclC, and HCC827EMT cells. As shown in Figure 5C, abemaciclib induced a reduction of phosphorylation of AKT protein, as already demonstrated in H460 and H1975 NSCLC cell lines [16]. Moreover, abemaciclib by inactivating AKT induced a significant dephosphorylation of MDM2 with the consequent accumulation of p53 protein. This mechanism occurred even if p53 was mutated [17]. In addition, we observed an accumulation of p21 protein. It is conceivable that this increase depended on abemaciclib-induced inhibition of c-Myc consequent to Rb hypo-phosphorylation. Indeed, c-Myc is known to negatively regulate p21 transcription through a direct binding to its promoter [18]. The increase of p21 levels might account for abemaciclib-mediated induction of senescence, being this protein a well-known positive regulator of senescence [19,20,21]. In addition, abemaciclib treatment induced an accumulation of cyclin D1, which, together with c-Myc inactivation, is considered as a marker associated to cellular senescence [22,23]. We then evaluated the effect of abemaciclib and abemaciclib plus osimertinib in long-term experiments (4 weeks) in the resistant PC9BRAFG469A and PC9T790MclC cells. The cells stopped their growth, as expected, and after 1 month all the cells were positive for β-galactosidase staining and there were no differences in the two conditions in term of cell number, as evaluated by crystal violet assay (Figure 5D).

### 2.4. Abemaciclib Combined with Osimertinib Prevents the Appearance of Osimertinib Resistance

In the last part of this study, we evaluated whether abemaciclib may prevent the development of acquired resistance to osimertinib in sensitive EGFR-mutated cells.

We first tested the efficacy of combining abemaciclib with osimertinib in PC9, HCC827, PC9T790M, and H1975 osimertinib-sensitive cell lines. Differently from resistant cells, the combination was more effective in inhibiting cell proliferation than osimertinib alone even if only at very low drug concentrations (Figure S2). This result is in agreement with the data obtained with lerociclib (G1T38), another CDK4/6 inhibitor, combined with osimertinib [24].

To evaluate the appearance of resistance, PC9, PC9T790M, and H1975 cells were plated in 24-well plates and treated with 500 nM osimertinib, a concentration close to the plasma level in patients treated with 80 mg/day dosage [14], in absence or presence of 500 nM abemaciclib, the steady state Cmax achieved in patients at a dose of 200 mg BID [25].

**Figure 6:**
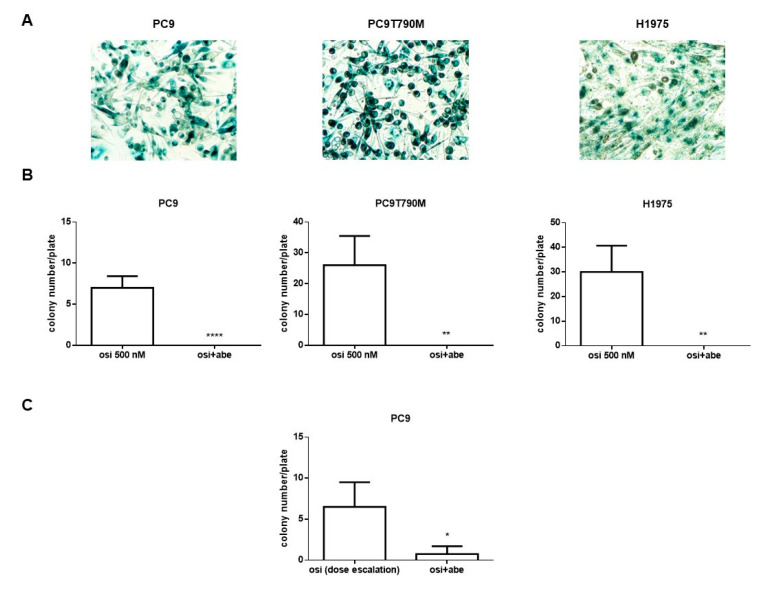
Effect of the combined treatment of abemaciclib and osimertinib on the acquisition of osimertinib resistance in osimertinib-sensitive cells. (**A**) PC9, PC9T790M, and H1975 cells were plated in 24-well plates and treated with 500 nM abemaciclib. After 2 weeks, senescent cells were quantified by SA-β-Gal staining (magnification 100×). (**B**) PC9, PC9T790M, and H1975 cells were plated in 24-well plates and treated with 500 nM osimertinib from the beginning in absence or in presence of 500 nM abemaciclib. Colony number was evaluated after 12 weeks for PC9 cells, 6 weeks for PC9T790M cells, and 4 weeks for H1975 cells. (**C**) PC9 cells were plated in 24-well plates and treated with increasing concentrations of osimertinib (starting from 25 nM to 500 nM) and after 12 weeks colony number was assessed (* *p* < 0.05, ** *p* < 0.01, **** *p* < 0.0001).

It is of note that sensitive cells treated with abemaciclib alone underwent senescence and in 2 weeks all the seeded cells were positive to β-galactosidase staining (Figure 6A). Treatment with osimertinib alone led to the inhibition of cell proliferation associated with cell death; resistant colonies emerged after 4 weeks for H1975 cells, after 6 weeks for PC9T790M cells, and after 12 weeks for PC9 cells. We did not find any colonies in the presence of the combined treatment in all three sensitive cell models tested throughout the entire experiments (Figure 6B).

In an additional experiment, PC9 cells were treated with increasing concentrations of osimertinib (starting from 25 nM up to 500 nM) in absence or in presence of 500 nM abemaciclib. The treatment with increasing doses of osimertinib alone led to the emergence of resistant colonies after 9 weeks, and abemaciclib was effective in postponing and reducing the emergence of acquired resistance to osimertinib. Indeed, as shown in Figure 6C only three colonies were present in all the plates after 12 weeks of combined treatment and these colonies were characterized by few (<than 20 cells) enlarged cells, which were not able to proliferate even after 14 days of drug removal.

These results suggest that a high concentration of osimertinib from the beginning, when combined with abemaciclib, is more effective than a strategy of dose increase, and completely prevents resistance. Altogether, our results indicate the potential value of this combined administration for mutated NSCLC patients in preventing osimertinib resistance.

## 3. Discussion

In this study we demonstrated that the CDK4/6 inhibitor abemaciclib alone or combined with osimertinib may be an effective strategy for NSCLC osimertinib-resistant patients progressed after either first- or second-line therapy. Interestingly, the combination is undergoing clinical evaluation in a phase II trial (ClinicalTrials.gov NCT04545710), started in September 2020, that has been evaluating abemaciclib combined with osimertinib in resistant EGFR mutated lung cancer patients post progression on osimertinib.

The CDK4/6-Cyclin D-Rb pathway is one of the most frequently dysregulated in cancer and more than 40% of human cancers show alterations in CDKs or cyclins. In NSCLC, cell-cycle gene alterations (CDK4/6 or CCND/E1 amplifications, CDKN2A loss or Rb mutations) have been associated with worse outcome [26,27]. In addition, in patients with advanced EGFR mutated NSCLC, co-alterations of cell cycle genes, such as CCND1/2, CCNE1, CDK4/6 were significantly associated with intrinsic resistance to osimertinib [28]. Alterations in genes encoding cell cycle regulators have been also reported in almost 10% of patients who progressed to osimertinib treatment either in second- or in first-line therapy [29,30].

In this study, we evaluated the activation of CDK4/6-Cyclin D-Rb pathway in NSCLC cell lines resistant to osimertinib, with acquired resistance to first-line (PC9BRAFG469A cell line) or second-line therapy (PC9T790MclA, PC9T790MclC and PC9T790MC797S cell lines) and intrinsically resistant (HCC827EMT and HCC827GR5 cell lines).

Rb has been found lost in 100% of NSCLC patients underwent histological transformation to small-cell lung cancer (SCLC) after gefitinib treatment [31], but no data are available on the phosphorylation status of Rb in patients with different mechanisms of resistance to EGFR-TKI. It has been reported that afatinib did not suppress Rb phosphorylation in afatinib-resistant PC9 cells [13], and, very recently, that p-Rb was increased in osimertinib-resistant H1975 cells (H1975OR), with an undefined resistance mechanism, in comparison with the parental ones [12].

Here, we demonstrated that all the resistant cell lines maintained Rb phosphorylation in the presence of osimertinib and were therefore sensitive to the cytostatic activity of the CDK4/6 inhibitor abemaciclib. In addition, excluding HCC827EMT, lacking p16 expression, all the other cell models express the protein and are sensitive to abemaciclib. These results are in agreement with previous findings indicating the expression of a functional Rb protein as a predictive biomarker of response to CDK4/6 inhibitors, but not with those showing the expression of p16^INK4a^ as a resistance factor to CDK4/6 inhibitors [32]. The resistant cell models did not show an increased sensitivity to abemaciclib as compared with the parental cells, already sensitive to the CDK4/6 inhibitor (IC_50_ lower than 1 µM). This result is in contrast with a very recent study reporting that in the H1975OR cell clone the IC_50_ for palbociclib was reduced from 12.94 (that is the IC_50_ value in the parental cells) to 4.776 µM [12].

Rb phosphorylation is a final step on which multiple genetic alterations converge to sustain cell proliferation during drug resistance and p-Rb may be considered as a biomarker of osimertinib-resistance and a potential target for therapeutic intervention. Therefore, targeting CDK4/6, the kinases responsible for Rb phosphorylation, may represent a new chance for patients progressing to osimertinib treatment, considering the complex patterns of resistance that render difficult to find a suitable targeted therapeutic approach.

The activity of abemaciclib was tested either as monotherapy or in combination with osimertinib, mimicking a clinical condition of removal or maintenance of osimertinib after disease progression. Abemaciclib alone inhibited cell growth, spheroid formation, and colony formation, and induced senescence in the resistant cell lines, without restoring the sensitivity to osimertinib. By contrast, a synergistic activity on cell proliferation has been very recently documented in H1975OR cell clone when treated with palbociclib combined with osimertinib [12]. The different cell model, the different range of responsiveness and the different drug may explain this discrepancy. Moreover, the authors themselves underlined the need to expand their study including more cell lines, such as resistant clones from PC9 and HCC827, to confirm whether the combination could actually overcome acquired resistance to osimertinib.

A relevant challenge in the clinic is the development of strategies to prevent the emergence of resistance during osimertinib treatment. This study provides a demonstration that in osimertinib sensitive cells the addition of abemaciclib to osimertinib can block the emergence of resistance. Data presented at the AACR 2019 Meeting [24] demonstrated in xenograft models that a combined treatment with lerociclib and osimertinib prevented the acquisition of resistance in HCC827-derived tumors and was able to revert resistance in tumors derived from HCC827/ER1 MET-amplified cells. The lack of in vivo studies represents a limitation of our work, and further animal validation is needed.

The strategy of a drug combination to be used in the clinic to postpone osimertinib-resistance has been evaluated either in preclinical studies or in clinical trials. We recently demonstrated the efficacy of osimertinib combined with pemetrexed or cisplatin in NSCLC PC9 and HCC827 cell lines and in PC9T790M nude mice xenografts [33]. In phase III studies, first-generation TKIs plus chemotherapy have led to promising results [34], and FLAURA 2 study (NCT04035486) is evaluating osimertinib plus chemotherapy versus osimertinib alone. The combination of osimertinib with the MEK inhibitor selumetinib was found to prevent EGFR-TKI resistance both in vitro and in vivo [35]. Other potential combinations that are being evaluated are osimertinib with anti-angiogenic agents (such as bevacizumab [36]), and with an AXL inhibitor [37].

All these results strongly suggest that osimertinib combinations could represent an interesting strategy for the future first-line treatment of EGFR-mutated NSCLC patients.

## 4. Materials and Methods

### 4.1. Cell Lines and Culture

The NSCLC cell line PC9 was kindly provided by Dr. P. Jänne (Dana-Farber Cancer Institute, Boston, MA, USA). PC9T790M cell clone was generated in our lab by exposing PC9 to increasing concentrations of gefitinib [38]. HCC827 and H1975 cell line were from ATCC (Manassas, VA, USA). PC9, HCC827 and H1975 are mutated for p53 (R248Q, V218del and R273H, respectively) and wild type for Rb (https://p53.iarc.fr/TP53GeneVariations.aspx; https://cancer.sanger.ac.uk/cell_lines) [39,40,41].

The resistant clones showed different mechanism of intrinsic or acquired resistance to osimertinib (Figure 1A): PC9BRAFG469A cells carrying a BRAF G469A mutation were generated in our lab by exposing PC9 cells to increasing concentration of osimertinib for 9 months [42]; PC9T790MclA cells were generated in our lab but with still undefined resistance mechanism. The clone was positive for EGFR exon 19 deletion and T790M mutation, negative for C797S, and amplification of MET or HER-2, or phenotypic transformation were not identified as potential mechanisms of resistance. NGS analysis did not reveal any acquired mutations [33]; PC9T790MclC cells with NRAS amplification were generated in our lab by exposing PC9T790M to osimertinib for 9 months [33]; PC9T790MC797S cells with C797S mutation were provided by Dr M. Mancini and originated by exposing PC9T790M cells to osimertinib [43]; HCC827EMT cells with epithelial to mesenchymal transition were generated in our lab by exposing HCC827 to gefitinib resulting also intrinsically resistant to osimertinib [38]; HCC827GR5 cells with MET amplification intrinsically resistant to osimertinib were provided by Dr. P. Jänne. Cells were cultured in RPMI-1640 (Life Technologies, Gaithersburg, MD, USA) medium supplemented with 10% fetal bovine serum (Invitrogen, Carlsbad, CA, USA) and maintained under standard cell culture conditions at 37 °C in a water-saturated atmosphere of 5% CO2 in air. Resistant cells were routinely cultured in the presence of 1 µM gefitinib (HCC827EMT, HCC827GR5) or 500 nM osimertinib (PC9BRAFG469A, PC9T790MclA, PC9T790MclC and PC9T790MC797S) to maintain a selection pressure during in vitro propagation.

### 4.2. Drug Treatment

Osimertinib was provided by AstraZeneca (Milan, Italy). Abemaciclib and palbociclib were from Selleckem (Houston, TX, USA). The drugs were dissolved in DMSO (Sigma Aldrich, St Louis, MO, USA). Final DMSO concentration in medium never exceeded 0.1% (v/v) and equal amounts of the solvent were added to control cells.

### 4.3. Analysis of Cell Proliferation, Cell Death and Cell Cycle

Cell proliferation and viability was evaluated by tetrazolium dye [3-(4,5-dimethylthiazol-2-yl)-2,5-diphenyltetrazolium bromide] (MTT) assay as previously described [44]. Cell death (assessed by Hoechst 33342 and propidium iodide dual staining and fluorescence microscopy analysis) and distribution of the cells in the cell cycle (determined by PI staining and flow cytometry analysis) were described elsewhere [45].

The nature of interaction between abemaciclib and osimertinib was calculated using the Bliss additivism model as previously described [46]. Briefly a theoretical dose-response curve was calculated for combined inhibition using the equation EBliss = EA + EB − EA * EB, where EA and EB are the percent of inhibition versus control obtained by osimertinib (A) and abemaciclib (B) alone and the EBliss is the percent of inhibition that would be expected if the combination was additive. If the combination effect is higher than the expected EBliss value the interaction is synergistic, while if the effect is lower, the interaction is antagonistic. Otherwise, the effect is additive and there is no interaction between drugs.

To further investigate the interaction between osimertinib and abemaciclib cells were treated with different concentrations of the two compounds using a TECAN D300e Digital Dispenser (TECAN, Switzerland). After 72 h, cell proliferation was assessed by crystal violet staining. Heatmap and statistics were generate using Combenefit on MATLAB [47].

### 4.4. Spheroid Generation

Spheroids were generated using LIPIDURE-COAT PLATE A-U96 (NOF Corporation, Tokyo, Japan) according to the manufacturer’s instruction and as described previously [48].

### 4.5. Colony Formation Assay

Cells were seeded in 6-well culture plates at a density of 5 × 10^3^ cells per well. Cells were incubated at 37 °C in 5% CO_2_ incubator and the medium was changed every 3 days, and at the end of the experiment, cells were fixed with ice-cold methanol, stained with 0.1% crystal violet (Sigma Aldrich). The unbound dye was removed by washing with water. The bound crystal violet was solubilized with 0.2% TritonX-100 in PBS and the absorbance of the solution was measured at a wavelength of 570 nm.

In the experiments testing resistance acquisition, 15 × 10^3^ cells were plated in 24 well-plates and after 4–12 weeks of treatment (depending on cell model) colonies were fixed, stained and counted. Colonies containing at least 20 cells were scored and data were given as colony number per 24-well plate.

### 4.6. Senescence Evaluation

The evaluation of Senescence Associated β-Galactosidase (SA-β-Gal) expression was performed using the Senescence β-Galactoside Staining kit (Cell Signaling Technology Inc., Beverly, MA, USA) as previously described [15]. The number of SA-β-Gal positive cells (blue stained) was evaluated by cell counting in four randomly chosen microscope fields (100× magnification).

### 4.7. Western Blot Analysis

Procedures for protein extraction, solubilization, and protein analysis by 1-D PAGE are described elsewhere [41]. Antibodies against p-Rb^ser780^, p-Rb^ser807/ser811^, Rb, Cyclin D1, p16^INK4a^, p-CDK6, CDK6, p-AKT^ser473^, AKT, pEGFR^tyr1068^, EGFR, p53, p-MDM2, p21, c-Myc, actin, and HRP-conjugated secondary antibodies was from Cell Signaling Technology; the chemiluminescence system (Immobilion^TM^ Western Chemiluminescent HRP Substrate) was from Millipore (Temecula, CA, USA). Reagents for electrophoresis and blotting analysis were from BIO-RAD (Hercules, CA, USA). The whole Western blots are shown in Figures S3–S5.

### 4.8. Statistical Analysis

Statistical analyses were carried out using GraphPad Prism version 6.0 software (GraphPad Software Inc., San Diego, CA, USA). Results are expressed as mean values ± standard deviations (SD). Differences between the mean values recorded for different experimental conditions were evaluated by Student’s *t*-test or by one-way ANOVA followed by Bonferroni’s post-test, and p values are indicated where appropriate in the figures and in their legends. Adjusted *p* values of less than 0.05 were considered significant.

## 5. Conclusions

For advanced NSCLC EGFR-mutated patients with T790M positive tumors progressing after second-line and for patients progressing after first-line osimertinib, chemotherapy remains the standard of care. Our preclinical data strongly support the ongoing clinical evaluation of abemaciclib in combination with osimertinib in osimertinib-resistant patients regardless of the type of resistance developed, additionally suggesting that abemaciclib might be equally effective as monotherapy. Moreover, the combination of osimertinib with abemaciclib strongly reduced the appearance of resistance and may represent a potential future strategy for the first-line therapy.

## Data Availability

The data presented in this study are available on request from the corresponding author.

## Supplementary Materials

The following are available online at https://www.mdpi.com/2072-6694/13/1/6/s1, Figure S1: Effect of palbociclib combined with osimertinib on cell proliferation of osimertinib-resistant cells; Figure S2: Effect of abemaciclib combined with osimertinib on cell proliferation of osimertinib-sensitive cells; Figure S3: whole Western blots of Figure 1; Figure S4: whole Western blots of Figure 4; Figure S5: whole Western blots of Figure 5.

## References

[1] Cross D.A.E., Ashton S.E., Ghiorghiu S., Eberlein C., Nebhan C.A., Spitzler P.J., Orme J.P., Finlay M.R.V., Ward R.A., Mellor M.J. (2014). AZD9291, an Irreversible EGFR TKI, Overcomes T790M-Mediated Resistance to EGFR Inhibitors in Lung Cancer. Cancer Discov..

[2] Ahn M.-J., Tsai C., Shepherd F.A., Bazhenova L., Sequist L.V., Hida T., Yang J.C.H., Ramalingam S.S., Mitsudomi T., Jänne P.A. (2019). Osimertinib in patients with T790M mutation-positive, advanced non–small cell lung cancer: Long-term follow-up from a pooled analysis of 2 phase 2 studies. Cancer.

[3] Mok T.S., Wu Y.L., Ahn M.J., Garassino M.C., Kim H.R., Ramalingam S.S., Shepherd F.A., He Y., Akamatsu H., Theelen W.S. (2017). Osimertinib or Platinum-Pemetrexed in EGFR T790M-Positive Lung Cancer. N. Engl. J. Med..

[4] Planchard D., Popat S., Kerr K., Novello S., Smit E.F., Faivre-Finn C., Mok T.S., Reck M., Van Schil P.E., Hellmann M.D. (2018). Metastatic non-small cell lung cancer: ESMO Clinical Practice Guidelines for diagnosis, treatment and follow-up. Ann. Oncol..

[5] Soria J.-C., Ohe Y., Vansteenkiste J., Reungwetwattana T., Chewaskulyong B., Lee K.H., Dechaphunkul A., Imamura F., Nogami N., Kurata T. (2018). Osimertinib in UntreatedEGFR-Mutated Advanced Non–Small-Cell Lung Cancer. N. Engl. J. Med..

[6] Ramalingam S.S., Vansteenkiste J., Planchard D., Cho B.C., Gray J.E., Ohe Y., Zhou C., Reungwetwattana T., Cheng Y., Chewaskulyong B. (2020). Overall Survival with Osimertinib in Untreated, EGFR-Mutated Advanced NSCLC. N. Engl. J. Med..

[7] Leonetti A., Sharma S., Minari R., Perego P., Giovannetti E., Gelsomino F. (2019). Resistance mechanisms to osimertinib in EGFR-mutated non-small cell lung cancer. Br. J. Cancer.

[8] Dickler M., Tolaney S.M., Rugo H.S., Cortés J., Diéras V., Patt D., Wildiers H., Hudis C.A., O’Shaughnessy J., Zamora E. (2017). MONARCH 1, A Phase II Study of Abemaciclib, a CDK4 and CDK6 Inhibitor, as a Single Agent, in Patients with Refractory HR+/HER2− Metastatic Breast Cancer. Clin. Cancer Res..

[9] Sledge G.W., Toi M., Neven P., Sohn J., Inoue K., Pivot X., Burdaeva O., Okera M., Masuda N., Kaufman P.A. (2017). MONARCH 2: Abemaciclib in Combination With Fulvestrant in Women With HR+/HER2− Advanced Breast Cancer Who Had Progressed While Receiving Endocrine Therapy. J. Clin. Oncol..

[10] Goetz M.P., Toi M., Campone M., Sohn J., Paluch-Shimon S., Huober J., Park I.H., Trédan O., Chen S.-C., Manso L. (2017). MONARCH 3: Abemaciclib As Initial Therapy for Advanced Breast Cancer. J. Clin. Oncol..

[11] Bonelli M., La Monica S., Fumarola C., Alfieri R. (2019). Multiple effects of CDK4/6 inhibition in cancer: From cell cycle arrest to immunomodulation. Biochem. Pharmacol..

[12] Qin Q., Li X., Liang X., Zeng L., Wang J., Sun L. (2020). CDK4/6 inhibitor palbociclib overcomes acquired resistance to third-generation EGFR inhibitor osimertinib in non-small cell lung cancer (NSCLC). Thorac. Cancer.

[13] Nie H., Zhou X., Shuzhang D., Nie C., Zhang X., Huang J. (2019). Palbociclib overcomes afatinib resistance in non-small cell lung cancer. Biomed. Pharmacother..

[14] Brown K., Comisar C., Witjes H., Maringwa J., De Greef R., Vishwanathan K., Cantarini M., Cox E. (2017). Population pharmacokinetics and exposure-response of osimertinib in patients with non-small cell lung cancer. Br. J. Clin. Pharmacol..

[15] Hafner M., Mills C.E., Subramanian K., Chen C., Chung M., Boswell S.A., Everley R.A., Liu C., Walmsley C.S., Juric D. (2019). Multiomics Profiling Establishes the Polypharmacology of FDA-Approved CDK4/6 Inhibitors and the Potential for Differential Clinical Activity. Cell. Chem. Biol..

[16] Naz S., Sowers A., Choudhuri R., Wissler M., Gamson J., Mathias A., Cook J.A., Mitchell J.B. (2018). Abemaciclib, a Selective CDK4/6 Inhibitor, Enhances the Radiosensitivity of Non-Small Cell Lung Cancer In Vitro and In Vivo. Clin. Cancer Res..

[17] Prives C., White E. (2008). Does control of mutant p53 by Mdm2 complicate cancer therapy?. Genes Dev..

[18] Wu S., Çetinkaya C., Muñoz-Alonso M.J., Von Der Lehr N., Bahram F., Beuger V., Eilers M., León J., Larsson L.-G. (2003). Myc represses differentiation-induced p21CIP1 expression via Miz-1-dependent interaction with the p21 core promoter. Oncogene.

[19] Bonelli M.A., Digiacomo G., Fumarola C., Alfieri R., Quaini F., Falco A., Madeddu D., La Monica S., Cretella D., Ravelli A. (2017). Combined Inhibition of CDK4/6 and PI3K/AKT/mTOR Pathways Induces a Synergistic Anti-Tumor Effect in Malignant Pleural Mesothelioma Cells. Neoplasia.

[20] Xu X., Lu Z., Qiang W., Vidimar V., Kong B., Kim J.J., Wei J.-J. (2014). Inactivation of AKT Induces Cellular Senescence in Uterine Leiomyoma. Endocrinology.

[21] Rufini A., Tucci P.J.F., Celardo I., Melino G. (2013). Senescence and aging: The critical roles of p53. Oncogene.

[22] Leontieva O.V., Blagosklonny M.V. (2013). CDK4/6-inhibiting drug substitutes for p21 and p16 in senescence: Duration of cell cycle arrest and MTOR activity determine geroconversion. Cell Cycle.

[23] Wu C.-H., Van Riggelen J., Yetil A., Fan A.C., Bachireddy P., Felsher D.W. (2007). Cellular senescence is an important mechanism of tumor regression upon c-Myc inactivation. Proc. Natl. Acad. Sci. USA.

[24] Freed D.M., Hall C.R., Strum J.C., Roberts P.J. (2019). CDK4/6 inhibition with lerociclib (G1T38) delays acquired resistance to targeted therapies in preclinical models of non-small cell lung cancer. Cancer Res..

[25] Patnaik A., Rosen L.S., Tolaney S.M., Tolcher A.W., Goldman J.W., Gandhi L., Papadopoulos K.P., Beeram M., Rasco D.W., Hilton J.F. (2016). Efficacy and Safety of Abemaciclib, an Inhibitor of CDK4 and CDK6, for Patients with Breast Cancer, Non-Small Cell Lung Cancer, and Other Solid Tumors. Cancer Discov..

[26] Le X., Puri S., Negrao M.V., Nilsson M.B., Robichaux J.P., A Boyle T., Hicks J.K., Lovinger K.L., Roarty E.B., Rinsurongkawong W. (2018). Landscape of EGFR-Dependent and -Independent Resistance Mechanisms to Osimertinib and Continuation Therapy Beyond Progression in EGFR-Mutant NSCLC. Clin. Cancer Res..

[27] Bhateja P., Chiu M., Wildey G., Lipka M.B., Fu P., Yang M.C.L., Ardeshir-Larijani F., Sharma N., Dowlati A. (2019). Retinoblastoma mutation predicts poor outcomes in advanced non small cell lung cancer. Cancer Med..

[28] Blakely C.M., Watkins T.B.K., Wu W., Gini B., Chabon J.J., E McCoach C., McGranahan N., A Wilson G., Birkbak N.J., Olivas V.R. (2017). Evolution and clinical impact of co-occurring genetic alterations in advanced-stage EGFR-mutant lung cancers. Nat. Genet..

[29] Papadimitrakopoulou V., Wu Y.-L., Han J.-Y., Ahn M.-J., Ramalingam S.S., John T., Okamoto I., Yang J.C., Bulusu K.C., Lauset G. (2018). LBA51 Analysis of resistance mechanisms to osimertinib in patients with EGFR T790M advanced NSCLC from the AURA3 study. Ann. Oncol..

[30] Ramalingam S.S., Cheng Y., Zhou C., Ohe Y., Imamura F., Cho B.C., Lin M., Majem M., Shah R., Rukazenkov Y. (2018). LBA50 Mechanisms of acquired resistance to first-line osimertinib: Preliminary data from the phase III FLAURA study. Ann. Oncol..

[31] Niederst M.J., Sequist L.V., Poirier J.T., Mermel C.H., Lockerman E.L., Garcia A.R., Katayama R., Costa C., Ross K.N., Moran T. (2015). RB loss in resistant EGFR mutant lung adenocarcinomas that transform to small-cell lung cancer. Nat. Commun..

[32] McCartney A., Migliaccio I., Bonechi M., Biagioni C., Romagnoli D., De Luca F., Galardi F., Risi E., De Santo I., Benelli M. (2019). Mechanisms of Resistance to CDK4/6 Inhibitors: Potential Implications and Biomarkers for Clinical Practice. Front. Oncol..

[33] La Monica S., Minari R., Cretella D., Flammini L., Fumarola C., Bonelli M., Cavazzoni A., Digiacomo G., Galetti M., Madeddu D. (2019). Third generation EGFR inhibitor osimertinib combined with pemetrexed or cisplatin exerts long-lasting anti-tumor effect in EGFR-mutated pre-clinical models of NSCLC. J. Exp. Clin. Cancer Res..

[34] Rebuzzi S.E., Alfieri R., La Monica S., Minari R., Petronini P.G., Tiseo M. (2020). Combination of EGFR-TKIs and chemotherapy in advanced EGFR mutated NSCLC: Review of the literature and future perspectives. Crit. Rev. Oncol..

[35] Eberlein C.A., Stetson D., Markovets A.A., Al-Kadhimi K.J., Lai Z., Fisher P.R., Meador C.B., Spitzler P., Ichihara E., Ross S.J. (2015). Acquired Resistance to the Mutant-Selective EGFR Inhibitor AZD9291 Is Associated with Increased Dependence on RAS Signaling in Preclinical Models. Cancer Res..

[36] Yu H.A., Schoenfeld A.J., Makhnin A., Kim R., Rizvi H., Tsui D., Falcon C., Houck-Loomis B., Meng F., Yang J.L. (2020). Effect of Osimertinib and Bevacizumab on Progression-Free Survival for Patients With Metastatic EGFR-Mutant Lung Cancers: A Phase 1/2 Single-Group Open-Label Trial. JAMA Oncol..

[37] Yochum Z.A., Cades J., Wang H., Chatterjee S., Simons B.W., O’Brien J.P., Khetarpal S.K., Lemtiri-Chlieh G., Myers K.V., Huang E.H.-B. (2019). Targeting the EMT transcription factor TWIST1 overcomes resistance to EGFR inhibitors in EGFR-mutant non-small-cell lung cancer. Oncogene.

[38] La Monica S., Madeddu D., Tiseo M., Vivo V., Galetti M., Cretella D., Bonelli M., Fumarola C., Cavazzoni A., Falco A. (2016). Combination of Gefitinib and Pemetrexed Prevents the Acquisition of TKI Resistance in NSCLC Cell Lines Carrying EGFR- Activating Mutation. J. Thorac. Oncol..

[39] Meder L., König K., Ozretić L., Schultheis A.M., Ueckeroth F., Ade C.P., Albus K., Boehm D., Rommerscheidt-Fuss U., Florin A. (2015). NOTCH, ASCL1, p53 and RB alterations define an alternative pathway driving neuroendocrine and small cell lung carcinomas. Int. J. Cancer.

[40] Lyu J., Yang E.J., Zhang B., Wu C., Pardeshi L., Shi C., Mou P.K., Liu Y., Tan K., Shim J.S. (2020). Synthetic lethality of RB1 and aurora A is driven by stathmin-mediated disruption of microtubule dynamics. Nat. Commun..

[41] Chou C.-W., Lin C.-H., Hsiao T.-H., Lo C.-C., Hsieh C.-Y., Huang C.-C., Sher Y.-P. (2019). Therapeutic effects of statins against lung adenocarcinoma via p53 mutant-mediated apoptosis. Sci. Rep..

[42] La Monica S., Minari R., Cretella D., Bonelli M., Fumarola C., Cavazzoni A., Galetti M., Digiacomo G., Riccardi F., Petronini P.G. (2019). Acquired BRAF G469A Mutation as a Resistance Mechanism to First-Line Osimertinib Treatment in NSCLC Cell Lines Harboring an EGFR Exon 19 Deletion. Target. Oncol..

[43] Mancini M., Gal H., Gaborit N., Mazzeo L., Romaniello D., Salame T.M., Lindzen M., Mahlknecht G., Enuka Y., Burton D.G. (2017). An oligoclonal antibody durably overcomes resistance of lung cancer to third-generation EGFR inhibitors. EMBO Mol. Med..

[44] Cavazzoni A., Alfieri R.R., Carmi C., Zuliani V., Galetti M., Fumarola C., Frazzi R., Bonelli M., Bordi F., Lodola A. (2008). Dual mechanisms of action of the 5-benzylidene-hydantoin UPR1024 on lung cancer cell lines. Mol. Cancer Ther..

[45] Fumarola C., La Monica S., Alfieri R.R., Borra E., Guidotti G.G. (2005). Cell size reduction induced by inhibition of the mTOR/S6K-signaling pathway protects Jurkat cells from apoptosis. Cell Death Differ..

[46] La Monica S., Galetti M., Alfieri R., Cavazzoni A., Ardizzoni A., Tiseo M., Capelletti M., Goldoni M., Tagliaferri S., Mutti A. (2009). Everolimus restores gefitinib sensitivity in resistant non-small cell lung cancer cell lines. Biochem. Pharmacol..

[47] Di Veroli G.Y., Fornari C., Wang D., Mollard S., Bramhall J.L., Richards F.M., Jodrell D.I. (2016). Combenefit: An interactive platform for the analysis and visualization of drug combinations. Bioinformatics.

[48] La Monica S., Cretella D., Bonelli M., Fumarola C., Cavazzoni A., Digiacomo G., Flammini L., Barocelli E., Minari R., Naldi N. (2017). Trastuzumab emtansine delays and overcomes resistance to the third-generation EGFR-TKI osimertinib in NSCLC EGFR mutated cell lines. J. Exp. Clin. Cancer Res..

