# Supplementary material for: Efficacy of the CDK4/6 Dual Inhibitor Abemaciclib in EGFR-Mutated NSCLC Cell Lines with Different Resistance Mechanisms to Osimertinib
Source: Cancers (Basel). 2020 Dec 22;13(1):6. doi: 10.3390/cancers13010006 (PMC7792603; doi:10.3390/cancers13010006)
Supplement: Supplementary file 1 [file cancers-13-00006-s001.zip › cancers-1031766-supplementary-final/cancers-1031766-suppl.pdf]

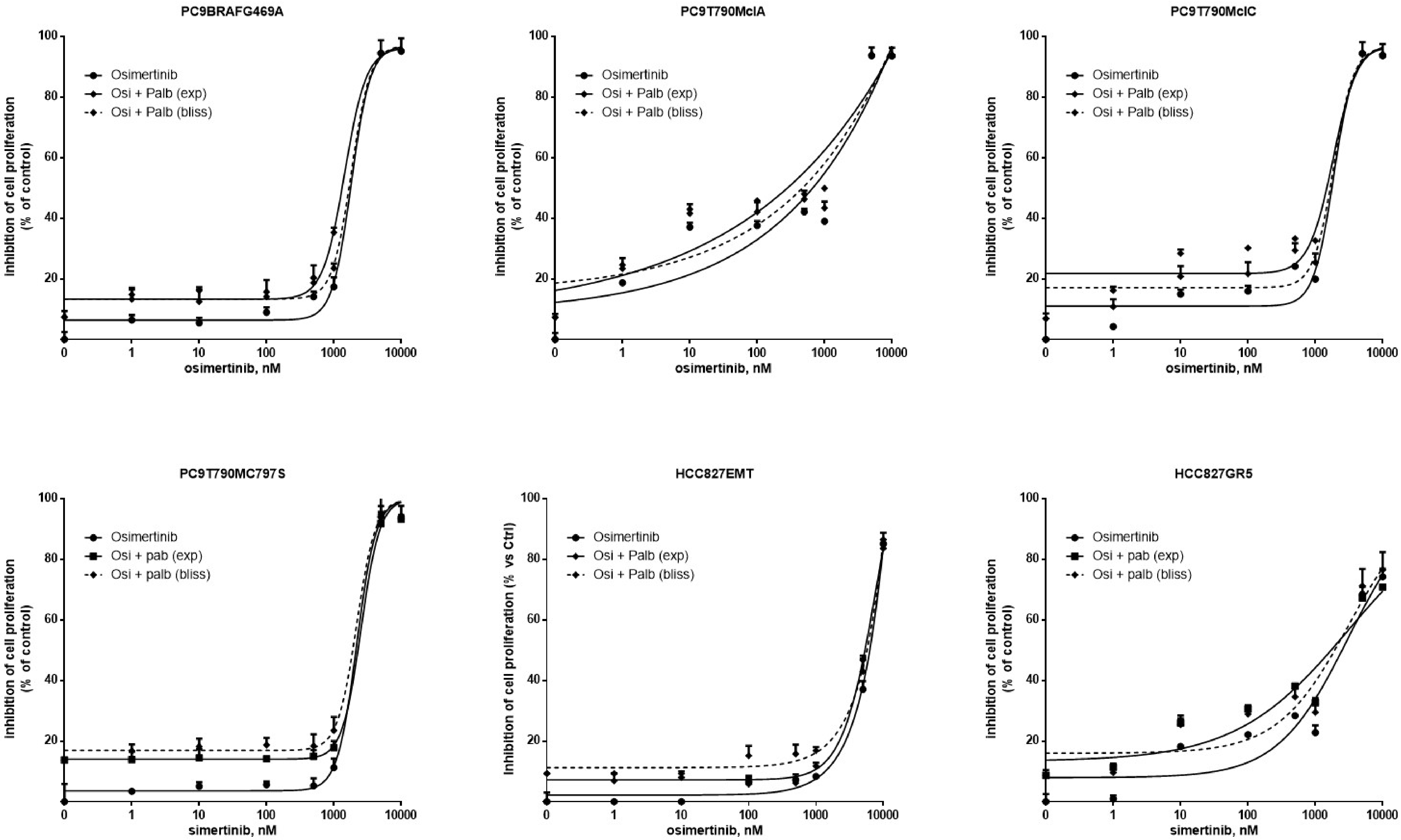

Figure S1: Effects of the combined treatment with palbociclib and osimertinib on cell proliferation. The indicated cells were treated with different concentrations of osimertinib in absence or in presence of 500nM palbociclib. After 72h, cell proliferation was assessed by MTT assay and the effect of the drug combination was evaluated using the Bliss interaction model. Data are expressed as percent inhibition vs control cells and are representative of at least two separate experiments.

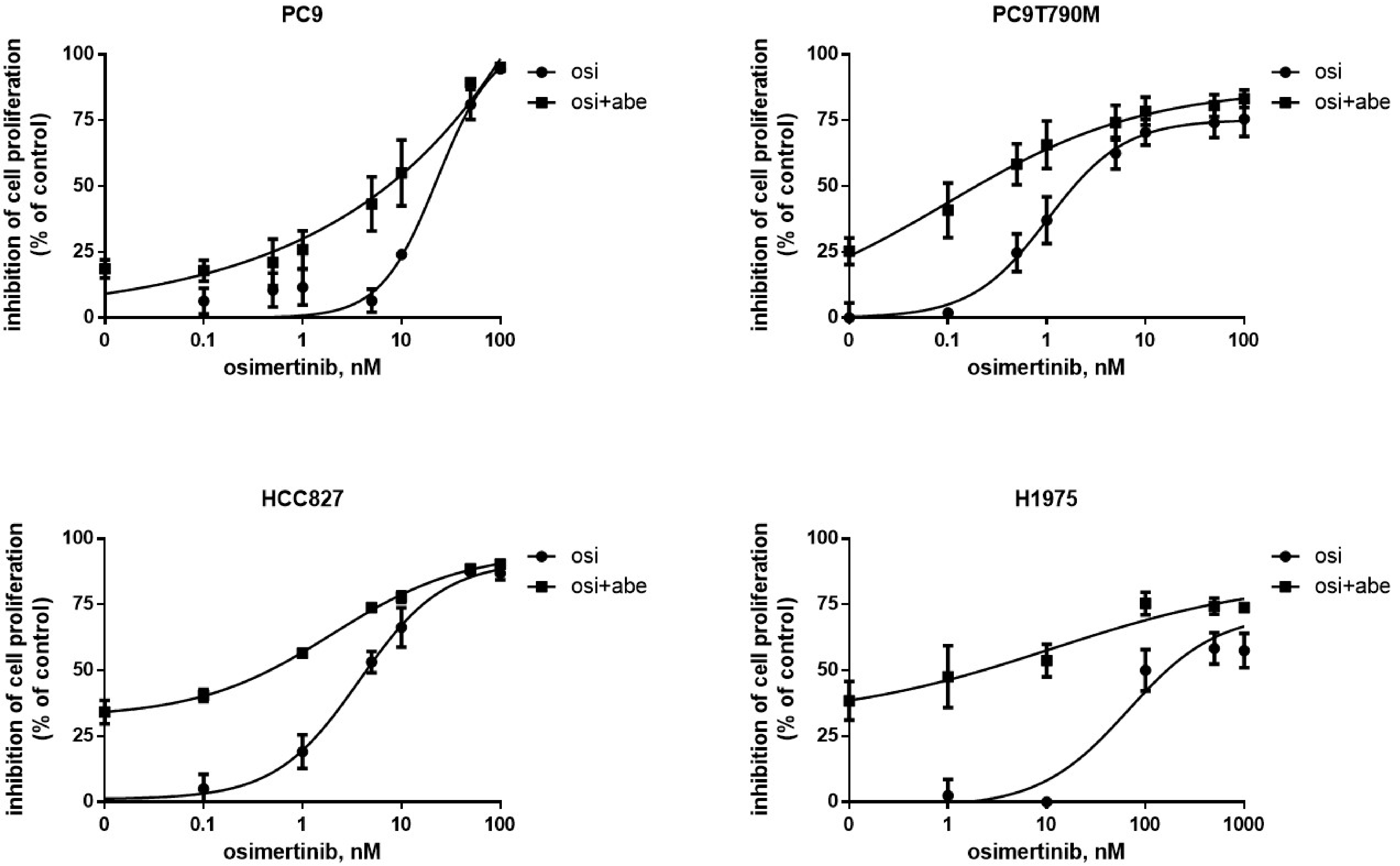

Figure S2: Effects of osimertinib alone or combined with abemaciclib on cell proliferation. The indicated cells were treated with different concentrations of osimertinib in absence or in presence of 500nM abemaciclib. After 72h, cell proliferation was assessed by MTT assay. Data are expressed as percent inhibition vs control cells and are representative of two separate experiments.
