# Supplementary material for: Efficacy of the CDK4/6 Dual Inhibitor Abemaciclib in EGFR-Mutated NSCLC Cell Lines with Different Resistance Mechanisms to Osimertinib
Source: Cancers (Basel). 2020 Dec 22;13(1):6. doi: 10.3390/cancers13010006 (PMC7792603; doi:10.3390/cancers13010006)

Full original blots used for Figure 1B. Each blot membrane was cut based on the standard band positions and then incubated with the corresponding antibodies.

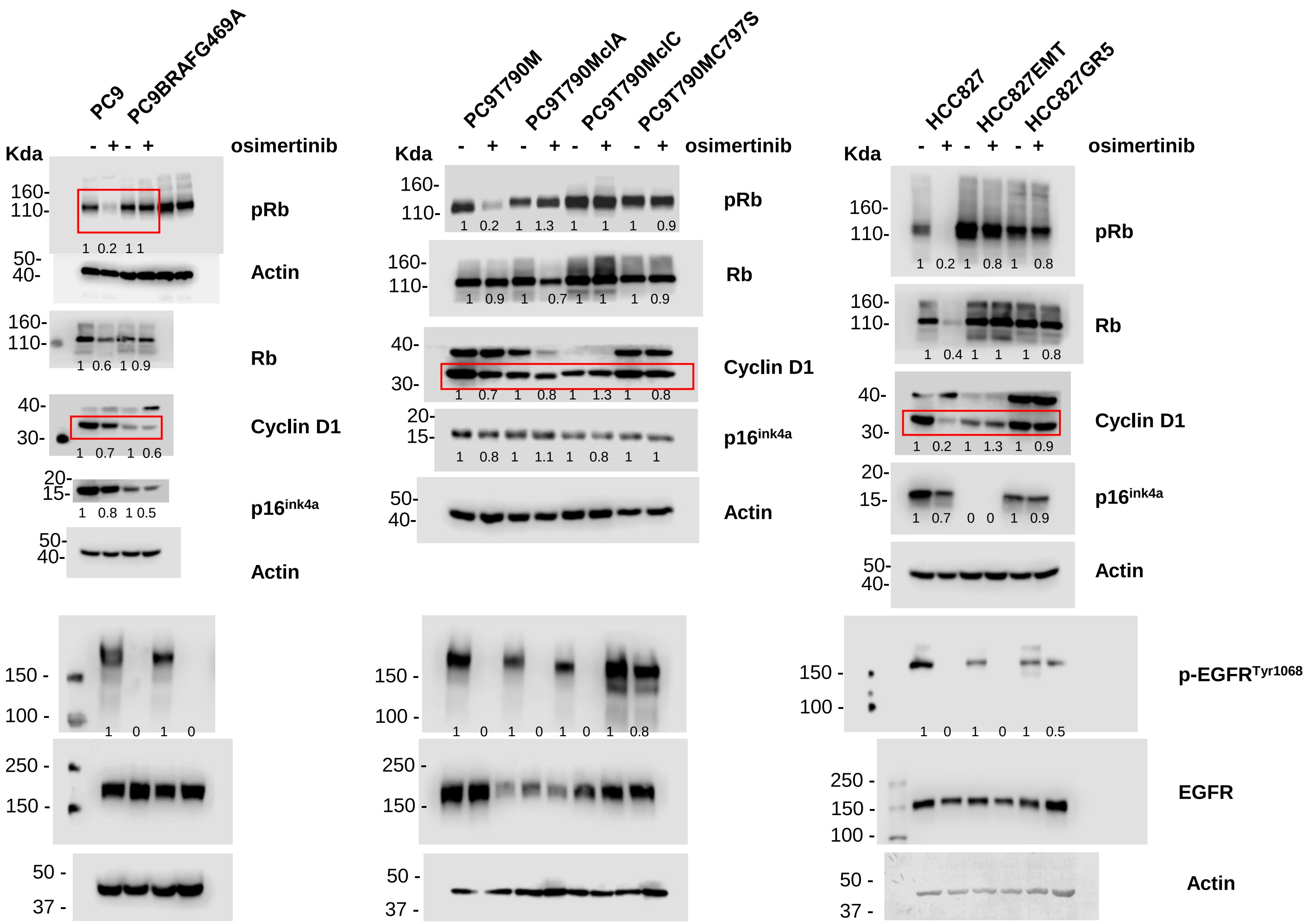

Full original blots used for Figure 4. Each blot membrane was cut based on the standard band positions and then incubated with the corresponding antibodies.

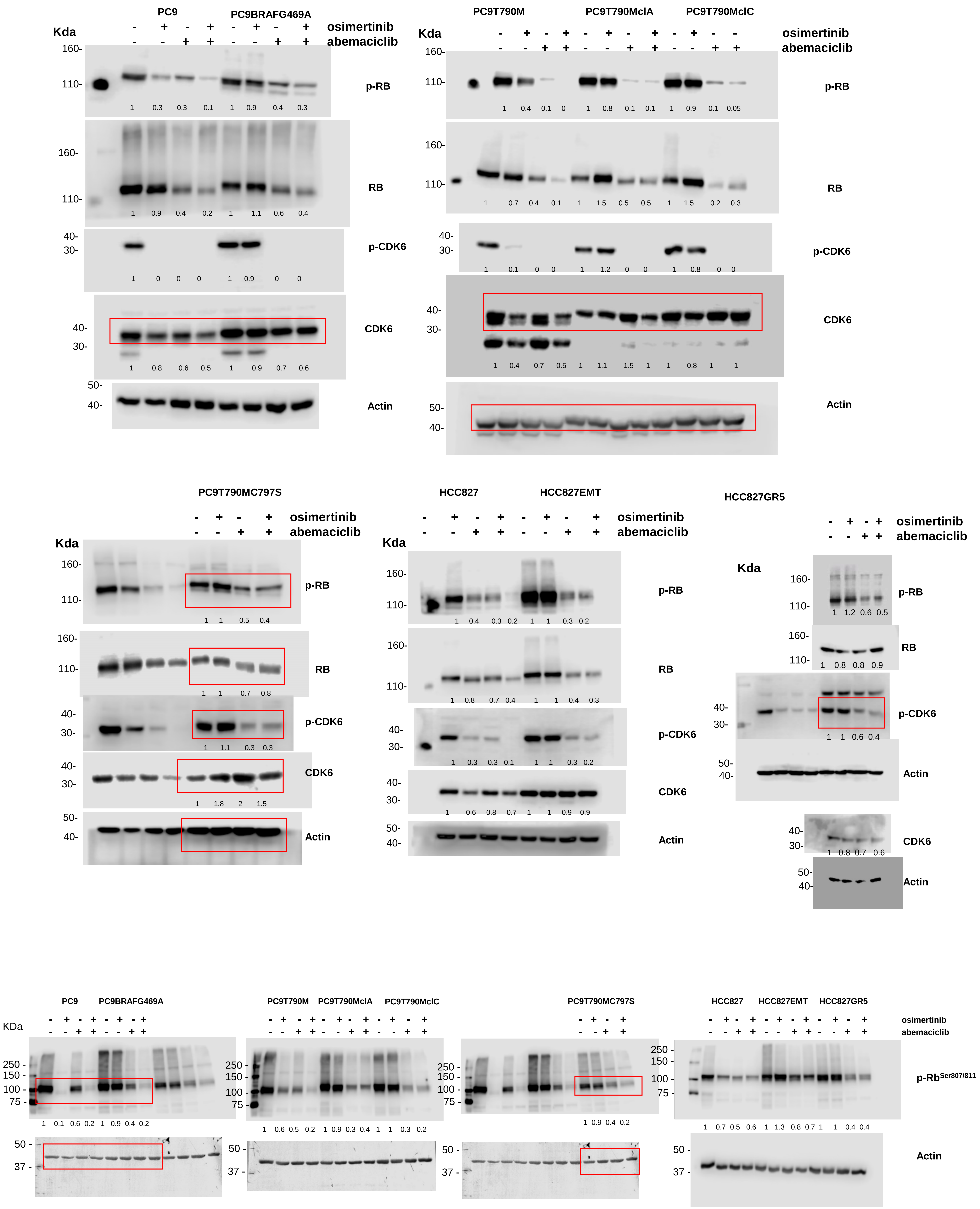

Full original blots used for Figure 5C. Each blot membrane was cut based on the standard band positions and then incubated with the corresponding antibodies.

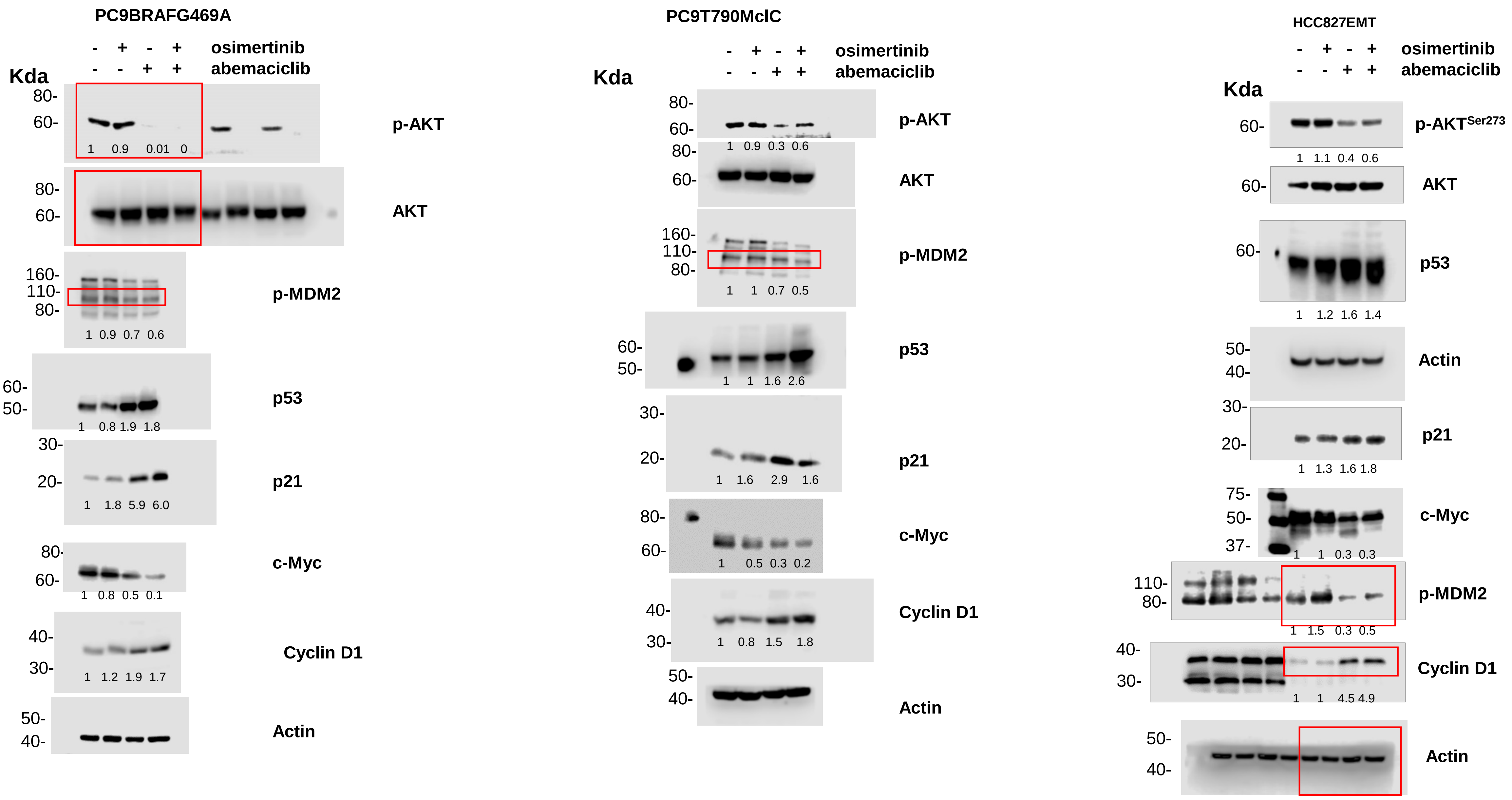

Supplement: Supplementary file 1 [file cancers-13-00006-s001.zip › cancers-1031766-supplementary-final/cancers-1031766-WB.pdf]
